# Comparison of different maxillary advancement protocols in patients with unilateral cleft lip and palate: a finite element analysis

**DOI:** 10.1007/s00784-024-05633-2

**Published:** 2024-04-20

**Authors:** Guleser Akdemir, Hande Gorucu-Coskuner

**Affiliations:** Hacettepe University, Ankara, Türkiye

**Keywords:** Face mask, Finite element analysis, Maxillary advancement, Unilateral cleft lip and palate

## Abstract

**Objectives:**

The aim of this study was to evaluate the stress distributions and possible amount of movement in the maxillofacial region resulting from different maxillary advancement protocols in patients with unilateral cleft lip and palate.

**Materials and methods:**

A unilateral cleft lip and palate model (CLP model) with Goslon score 4 was created for finite element analysis. Three different protocols were compared: Group 1: usage of a face mask with elastics placed at a 30? angle to the occlusal plane over a conventional acrylic plate; Group 2: usage of a face mask with elastics placed at a 30? angle to the occlusal plane over miniplates placed in the infrazygomatic crest region; Group 3: usage of elastic from the menton plate placed in the mandible to the infrazygomatic plates in the maxilla.

**Results:**

Dental effects were greater in the maxillary protraction protocol with a face mask over a conventional acrylic plate (Von Misses Stress Values; Group 1?=?cleft side:0.076, non-cleft side:0.077; Group 2?=?cleft side:0.004, non-cleft side: 0.003; Group 3?=?cleft side:0.0025; non-cleft side:0.0015), whereas skeletal effects were greater in maxillary protraction protocols with face mask using skeletal anchorage (Von Misses Stress Values; Group 1:0.008; Group 2:0.02; Group 3:0.0025). The maximum amount of counterclockwise rotation of the maxilla as a result of protraction was observed in traditional acrylic plate face mask protocol, and the minimum amount was observed by using elastics between infrazygomatic plates and menton plate.

**Conclusions:**

In individuals with unilateral cleft lip and palate with Goslon score 4, it was observed that the skeletally anchored face mask caused more skeletal impact and displacement than both the traditional acrylic plate face mask model and the pure skeletally supported maxillary protraction model.

**Clinical relevance:**

When planning maxillary protraction treatment in patients with cleft lip and palate, it should be considered that more movement in the sagittal plane might be expected on the cleft side than the non-cleft side, and miniplate and screws on the cleft side are exposed to more stress when using infrazygomatic plates as skeletal anchorage.

## Background

In individuals with complete cleft lip and palate, maxillary growth is often compromised by the restrictive forces resulting from the lip and palate repair [1]. As a result, individuals with cleft lip and palate often show a Class III skeletal pattern associated with anterior crossbite. Over the years, the most common treatment for mild maxillary retrusion in individuals with complete cleft lip and palate has been the usage of face mask for maxillary protraction [2]. Although favorable changes have been reported with face mask treatment, limited orthopedic effects can be achieved according to the severity of maxillary retrusion in individuals with unilateral cleft lip and palate [3, 4]. In addition, it may cause side effects such as molar mesialisation and maxillary incisor proclination in the maxillary dental arch [5]. Therefore, the most accepted treatment protocol for moderate or severe maxillary retrusion is maxillary advancement with orthognathic surgery at the end of the growth period [6]. The disadvantage of this protocol is that patients with unilateral cleft lip and palate, who usually exhibit facial dysmorphology, spend their childhood and adolescence without treatment [7].

Some studies have reported that the usage of modified surgical miniplates for skeletal anchorage with face mask treatment maximizes the orthopedic effects [8?^10^]. More recently, De Clerk et al. [11] and Nguyen et al. [12] introduced the Bone Anchored Maxillary Protraction (BAMP) protocol, which involves the placement of modified surgical miniplates in both maxilla and mandible, and usage of class III intermaxillary elastics over these plates. In this protocol, it was argued that pure bone-anchored orthopedic forces applied with intermaxillary elastics over miniplates can increase midfacial growth of patients with maxillary retrusion and prevent unwanted vertical growth in the lower facial region, unlike the traditional face mask protocol applied over the acrylic plate.

Mars et al. [13] published a simple method for scoring treatment outcomes in patients with unilateral cleft lip and palate called the GOSLON (Great Ormond Street, London and Oslo) criterion in which the possible treatment outcomes are displayed on models and scored by experienced assessors. According to this criterion, cases with Goslon score 4 are at the limit that can be treated orthopedically to correct the skeletal disorder; however, orthognathic surgical treatment might be required in adulthood if facial growth is not appropriate or if early treatment cannot be performed successfully. There is a lack of knowledge in the literature on the effects of traditional and skeletal maxillary advancement protocols in unilateral cleft lip and palate patients with Goslon score 4. There are limited number of finite element method studies on maxillary advancement treatment of children with cleft lip and palate, in which Goslon scores had not been mentioned [14?^16^]. Besides, to the best of our knowledge, no study has compared different protraction methods in cleft lip and palate patients.

The aim of this study was to evaluate the stress distributions and the amount of possible movement in the maxillofacial region caused by different maxillary advancement protocols in patients with unilateral cleft lip and palate using finite element analysis.

## Materials and methods

Institutional Review Board (IRB) of the Hacettepe University reviewed the study protocol and granted ethical approval (IRB No: GO 22/193). A cone-beam computed tomography image of an 11-year-old patient with unilateral cleft lip and palate and Goslon score 4 was used for the creation of the skull model used in the study. The arrangement of the three-dimensional network structure and its transformation into a mathematically appropriate solid network structure, the creation of three-dimensional finite element analysis models and the finite element analysis process were carried out on HP workstations with INTEL Xeon E-2286 processors with a clock speed of 2.40?GHz and 64 GB ECC memory.

The .stl model was obtained from the tomography data using 3DSlicer software. Reverse engineering and three-dimensional CAD activities were carried out with ANSYS Spaceclaim software, solid models were made suitable for the analysis environment, and optimized mesh generation activities were performed with ANSYS Workbench software; LS-DYNA solver was used for the solution of the finite element models created.

The trabecular bone was obtained by taking the inner surface of the three-dimensional mandibular cortical bone with adjusted thickness as reference. Periodontal ligaments in appropriate thickness were modelled with reference to the outer surface of the teeth. Sutures were created by performing ?boolean? operation between the bone segments with contact surfaces. The acrylic plate, infrazygomatic plate, face mask, menton plate and miniscrews were modelled in ANSYS Spaceclaim software based on the dimensions in the product catalogue (Fig.?1).

**Fig. 1:**
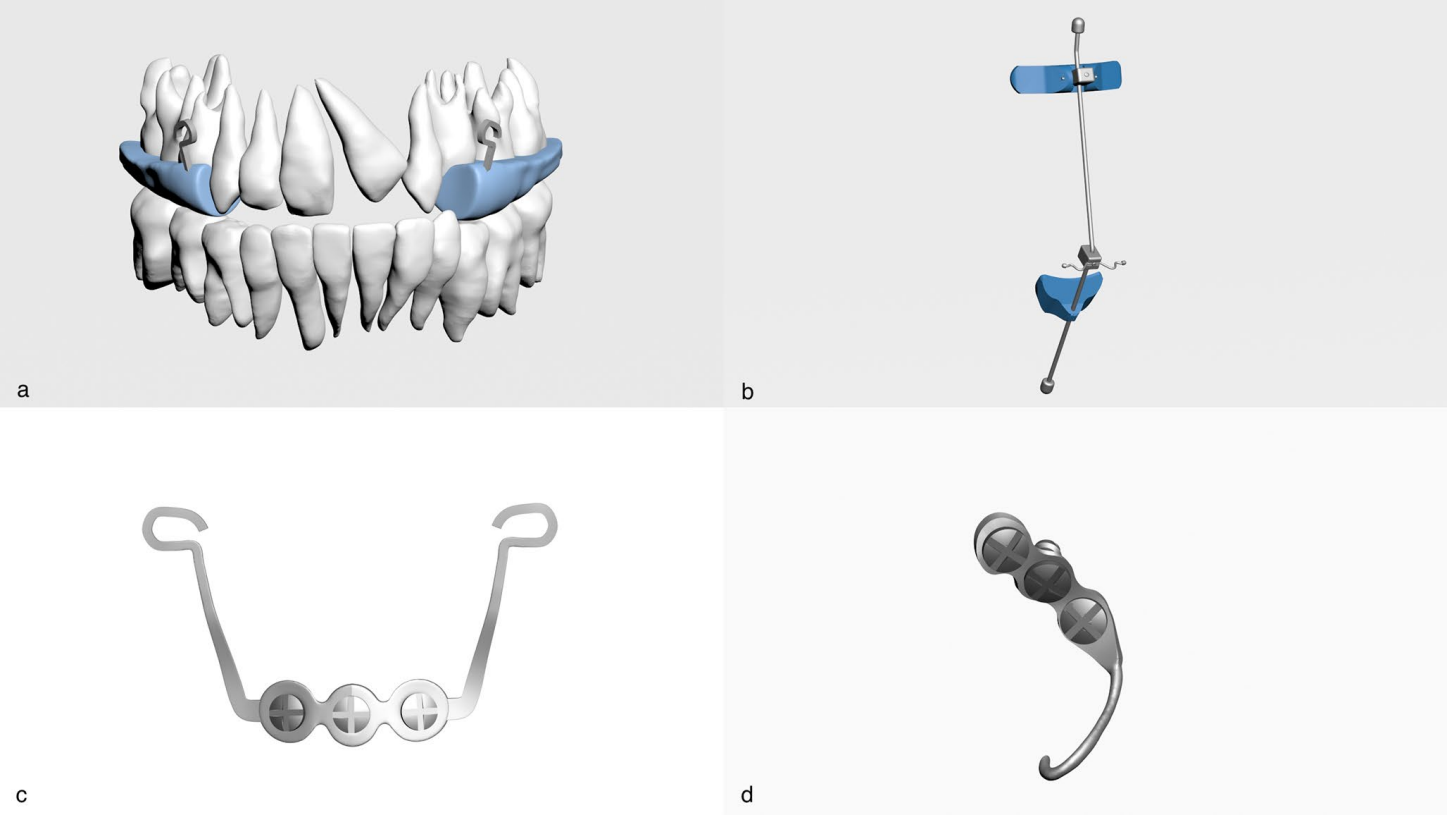
(**a**) acrylic plate, (**b**) face mask, (**c**) menton plate, (**d**) infrazygomatic plate

The acrylic plate was placed in each model to open the occlusion. Similar to the clinical conditions, acrylic parts were connected to each other with 0.043 inch stainless steel wire passing through the palatal region (Fig.?1). All prepared models were placed in the correct coordinates in 3D space in ANSYS Spaceclaim software and the modelling process was completed. The material properties given as elastic modulus and Poisson?s ratio were defined numerically (Table?1).

**Table 1:** Material properties of the analyzed model

	Elastic modulus [MPa]	Poisson?s ratio
**Cortical Bone**	13,700	0.30
**Trabecular Bone**	1370	0.30
**Tooth**	20,700	0.30
**Periodontal Ligament**	500	0.49
**Suture**	10	0.49
**Titanium Alloy**	114,000	0.3
**Stainless Steel**	193,000	0.3
**Acrylic**	15	0.35

Three different mechanics were compared in the unilateral cleft lip palate model (CLP model), as described below:

Group 1: The first mechanic was usage of a face mask with elastics placed at a 30? angle to the occlusal plane from a conventional acrylic plate (Fig.?2). The elastic forces were applied 500?g per side.

**Fig. 2:**
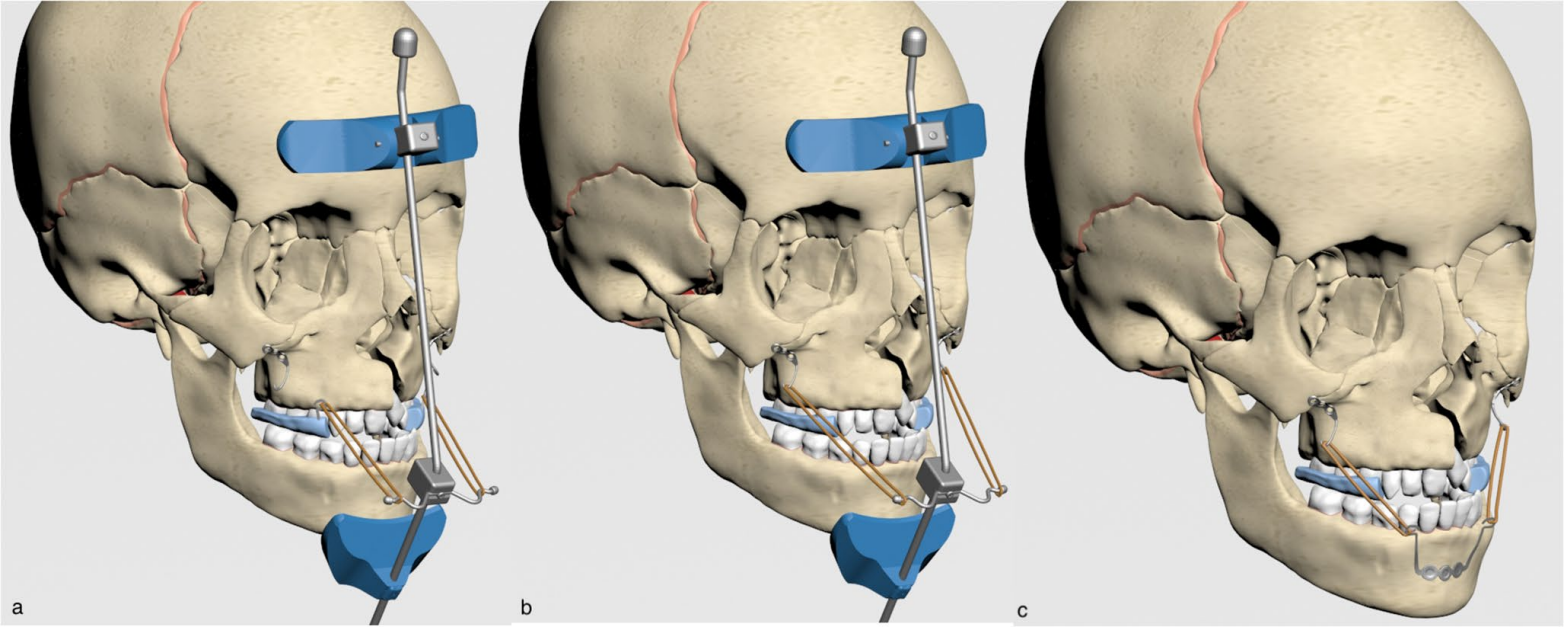
Mechanics used in the present study (**a**) face mask with elastics placed at a 30? angle to the occlusal plane from a conventional acrylic plate, (**b**) face mask with elastics placed at a 30? angle to the occlusal plane from miniplates placed in the infrazygomatic crest area, (**c**) elastics from the menton plate placed in the mandible to the infrazygomatic plates in the maxilla

Group 2: The second mechanic was usage of a face mask with elastics placed at a 30? angle to the occlusal plane from miniplates placed in the infrazygomatic crest area (Fig.?2). Three titanium miniplates of 1.2?mm profile thickness were placed in the infrazygomatic crest region of the maxilla, bilaterally, and these plates were secured with 3 titanium miniscrews of 5?mm length and 2.3?mm diameter. The elastic forces were applied 500?g per side, extending from miniplates to face mask.

Group 3: In BAMP protocol (Fig.?2), three titanium miniplates of 1.2?mm profile thickness were used. They were placed in the infrazygomatic crest region of the maxilla bilaterally and mandibular anterior region, and these plates were secured with 3 titanium miniscrews of 5?mm length and 2.3?mm diameter.

When the infrazygomatic plates and menton plates were placed in the most appropriate anatomical regions, the angle of the applied elastics was calculated as 40?. The elastic forces were applied 250?g per side, and elastics were used from the menton plate placed in the mandible to the infrazygomatic plates in the maxilla.

The models were fixed at the nodal points in the foramen magnum region by restricting all degrees of freedom to prevent movement in all three axes. A total of 3 linear static analyses were performed for 3 models under the specified force and boundary conditions. The number of elements and nodes for different analysis models are given in Table?2.

**Table 2:** Number of elements and nodes for different models used in the study

	Total Number of nodes	Total Number of elements
Acrylic plaque?+?Face mask	2,243,550	10,814,380
Infrazygomatic plate?+?Face mask	2,245,054	10,826,364
Infrazygomatic plate?+?Menton plate	2,183,795	10,516,826

In order to be able to perform analyses and obtain accurate results in the mathematical models created, the surface relations of the parts forming the model with each other must be defined in the analysis program. For this purpose, in all study models, the connection of all parts with contact between them was defined as ?bonded type contact?. This approach is based on the assumption that the parts move with full correlation during movement. Since the values obtained are the result of mathematical calculations without variance, statistical analyses cannot be performed. The aim was to carefully analyze and interpret the obtained values and stress distributions.

## Results

The changes occurred in the maxillary protraction models with three different anchorage methods were evaluated using Von Misses stress (Table?3) and displacement values (Tables?4, 5 and 6). Nasomaxillary complex and alveolar bone, cleft region, nasomaxillary suture, frontomaxillary suture, frontonasal suture, zygomaticomaxillary suture, zygomaticotemporal suture, zygomaticofrontal suture, maxillary dentition, infrazygomatic plates, menton plates, miniscrews and temporomandibular region were analyzed in this study.

**Table 3:** Von Misses stress values (MPa)

	Maxillary Protraction Model with Face Mask over Traditional Acrylic Plate		Maxillary Protraction Model with Face Mask over Infrazygomatic plate		Maxillary Protraction Involving the Use of Infrazygomatic Plates and Elastic Over Menton plate Model		
	Right Profile (Non-cleft side)	Left Profile (Cleft side)	Right Profile (Non-cleft side)	Left Profile (Cleft side)	Right Profile (Non-cleft side)	Left Profile (Cleft side)	
**Posterior Alveolar Region**	0.077	0.076	0.003	0.004	0.0015	0.0025	
**Nasomaxillary Suture (Mpa)**	0.022	0.061	0.032	0.092	0.013	0.022	
**Frontomaxillary** **Suture (Mpa)**	0.039	0.050	0.057	0.070	0.010	0.0065	
**Zygomaticomaxillary** **Suture (Mpa)**	0.038	0.039	0.058	0.062	0.034	0.036	
**Zygomaticotemporal** **Suture (Mpa)**	0.159	0.065	0.187	0.067	0.1055	0.0455	
**Zygomaticofrontal** **Suture (Mpa)**	0.075	0.114	0.095	0.156	0.038	0.0575	
**Midpalatal Suture** **(Mpa)**	0.001		0.002		0.00075		
**Frontonasal Suture** **(Mpa)**	0.119		0.135		0.039		

**Table 4:** Displacement values (mm) (X axis)

	Maxillary Protraction Model with Face Mask over Traditional Acrylic Plate		Maxillary Protraction Model with Face Mask over Infrazygomatic plate		Maxillary Protraction Involving the Use of Infrazygomatic Plates and Elastic Over Menton plate Model		
	Right Profile (Non-cleft side)	Left Profile (Cleft side)	Right Profile (Non-cleft side)	Left Profile (Cleft side)	Right Profile (Non-cleft side)	Left Profile (Cleft side)	
**Posterior Alveolar Region**	-4.054E-04	3.525E-05	-7.530E-05	-2.110E-04	3.507E-04	-4.352E-04	
**Nasomaxillary Suture (Mpa)**	-1.856E-04	-1.827E-04	-1.995E-04	-1.912E-04	-0.795E-04	-0.76E-04	
**Frontomaxillary Suture (Mpa)**	-1.564E-04	-1.491E-04	-1.678E-04	-1.598E-04	-0.674E-04	-0.662E-04	
**Zygomaticomaxillary Suture (Mpa)**	-3.385E-04	-3.668E-05	-1.542E-04	-2.513E-04	1.319E-04	-2.84E-04	
**Zygomaticotemporal Suture (Mpa)**	1.010E-03	-7.692E-04	1.144E-03	-8.210E-04	2.175E-04	0.988E-04	
**Zygomaticofrontal Suture (Mpa)**	-1.907E-04	-6.182E-05	-2.009E-04	-8.013E-05	-4.259E-05	-0.795E-04	
**Midpalatal Suture (Mpa)**	-4.038E-04		-1.876E-04		-0.745E-04		
**Frontonasal Suture (Mpa)**	-1.611E-04		-1.750E-04		-0.702E-04		

**Table 5:** Displacement values (mm) (Y axis)

	Maxillary Protraction Model with Face Mask over Traditional Acrylic Plate		Maxillary Protraction Model with Face Mask over Infrazygomatic plate		Maxillary Protraction Involving the Use of Infrazygomatic Plates and Elastic Over Menton plate Model		
	Right Profile (Non-cleft side)	Left Profile (Cleft side)	Right Profile (Non-cleft side)	Left Profile (Cleft side)	Right Profile (Non-cleft side)	Left Profile (Cleft side)	
**Posterior Alveolar Region**	-2.582E-03	-2.643E-03	-2.780E-03	-2.901E-03	-2.573E-04	-3.401E-04	
**Nasomaxillary Suture (Mpa)**	9.696E-04	9.271E-04	1.181E-03	1.126E-03	3.903E-05	2.836E-05	
**Frontomaxillary Suture (Mpa)**	1.227E-03	1.176E-03	1.475E-03	1.423E-03	0.689E-04	0.562E-04	
**Zygomaticomaxillary Suture (Mpa)**	-1.051E-03	-1.295E-03	-1.228E-03	-1.481E-03	-1.744E-04	-2.868E-04	
**Zygomaticotemporal Suture (Mpa)**	9.218E-04	-3.051E-04	9.322E-04	-5.202E-04	2.475E-04	4.216E-04	
**Zygomaticofrontal Suture (Mpa)**	1.721E-03	1.504E-03	1.936E-03	1.719E-03	1.088E-04	0.54E-04	
**Midpalatal Suture (Mpa)**	-2.192E-03		3.036E-05		-0.639E-04		
**Frontonasal Suture (Mpa)**	1.342E-03		1.595E-03		0.789E-04		

**Table 6:** Displacement values (mm) (Z axis)

	Maxillary Protraction Model with Face Mask over Traditional Acrylic Plate		Maxillary Protraction Model with Face Mask over Infrazygomatic plate		Maxillary Protraction Involving the Use of Infrazygomatic Plates and Elastic Over Menton plate Model		
	Right Profile (Non-cleft side)	Left Profile (Cleft side)	Right Profile (Non-cleft side)	Left Profile (Cleft side)	Right Profile (Non-cleft side)	Left Profile (Cleft side)	
**Posterior Alveolar Region**	3.452E-03	3.128E-03	3.939E-03	3.568E-03	1.336E-04	0.958E-04	
**Nasomaxillary Suture (Mpa)**	4.171E-03	4.180E-03	4.817E-03	4.837E-03	3.55E-04	3.563E-04	
**Frontomaxillary Suture (Mpa)**	3.608E-03	3.591E-03	4.155E-03	4.145E-03	2.792E-04	2.812E-04	
**Zygomaticomaxillary Suture (Mpa)**	3.428E-03	3.495E-03	3.814E-03	3.912E-03	2.557E-05	3.568E-05	
**Zygomaticotemporal Suture (Mpa)**	1.638E-03	1.497E-03	1.750E-03	1.642E-03	3.896E-04	2.626E-04	
**Zygomaticofrontal Suture (Mpa)**	3.382E-03	3.414E-03	3.820E-03	3.890E-03	2.061E-04	2.474E-04	
**Midpalatal Suture (Mpa)**	3.372E-03		3.920E-03		2.657E-04		
**Frontonasal Suture (Mpa)**	4.019E-03		4.668E-03		3.268E-04		

In Group 1, the Von Misses stress was seen in the alveolar bone around teeth (cleft side: 0.076; non-cleft side 0.077), the lateral walls of the alveolar processes (cleft side:0.039; non-cleft side:0.038), and the pterygomaxillary suture (cleft side: 0.114; non-cleft side:0.257). In Group 2, stress was observed in the lateral area of the zygomatic process (cleft side:0.067; non-cleft side:0.187), the infrazygomatic region (cleft side:0.062; non-cleft side:0.058), and the pterygomaxillary suture (cleft side: 0.099; non-cleft side:0.25). The intense stress seen around the infrazygomatic plates decreased gradually while showing a peripheral distribution. In Group 3, the areas with the highest stress were the infrazygomatic area where the plates were applied (cleft side:0.036; non-cleft side:0.034), the lateral parts of the zygomatic bone (cleft side:0.045; non-cleft side:0.105) and the lateral nasal walls (cleft side:0.006; non-cleft side:0.0025). Tension forces decreased by distributing from these three areas to the surrounding structures (Fig.?3).

**Fig. 3:**
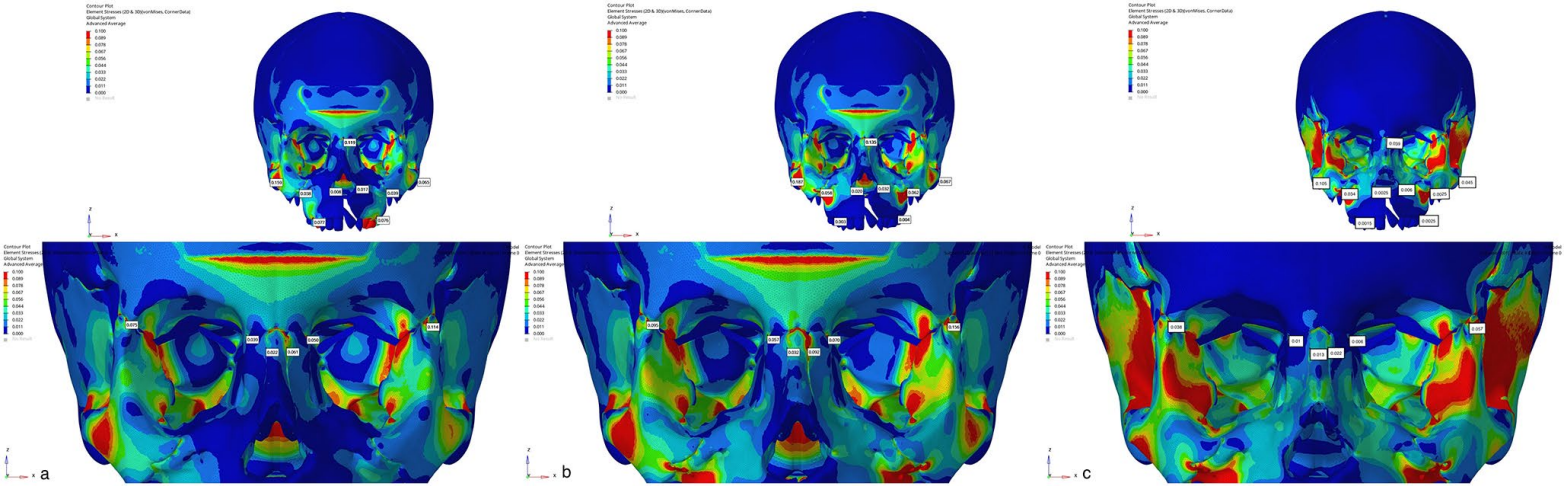
Frontal images of Von Misses stress values in maxillary protraction models with **a**. Face mask over acrylic plates, **b**. face mask over infrazygomatic plates and **c**. use of elastic over infrazygomatic and menton plates for CLP model

When the displacement values were analyzed in the axial plane (X-axis), expansion was observed on the cleft side in Group 1, while contraction was observed on the cleft side in other models. On the other hand, in Group 3, narrowing was observed on the non-cleft side, while expansion was observed in other groups. The highest displacement in the sagittal direction (Y-axis) was observed in Group 2, especially more on the cleft side. In this region, the highest displacement was mostly observed in the alveolar bone and in the peripheral structures surrounding the maxillary posterior teeth and gradually decreased towards the lateral nasal walls. In all models, a higher amount of movement was observed in the sagittal plane on the cleft side compared to the non-cleft side. In the vertical direction (Z axis), the maximum displacement was observed as the movement of the anterior maxillary region to the superior direction in the model in Group 2. The least movement in the vertical direction was observed in Group 3 (Figs.?4, 5 and 6).

**Fig. 4:**
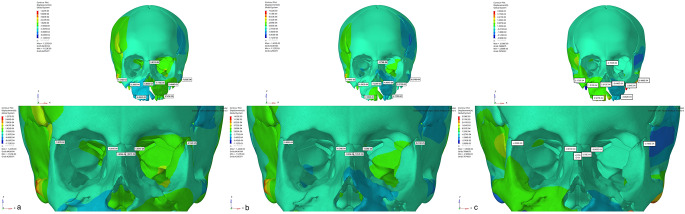
Frontal images (X Axis) of the displacement values in the maxillary protraction. models with the use of (**a**) Face mask over acrylic plates, (**b**) face mask over infrazygomatic plates and (**c**) elastic over infrazygomatic and menton plates for the CLP model

**Fig. 5:**
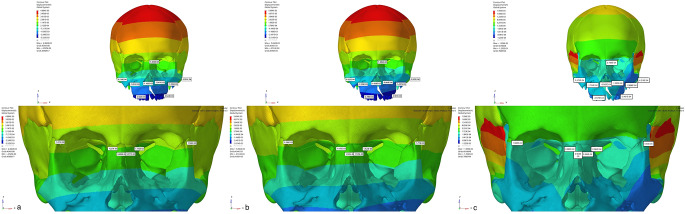
Frontal images of the displacement values in the maxillary protraction models with the use of (**a**) Face mask over acrylic plates, (**b**) face mask over infrazygomatic plates and (**c**) elastic over infrazygomatic and menton plate in the CLP model (Y Axis)

**Fig. 6:**
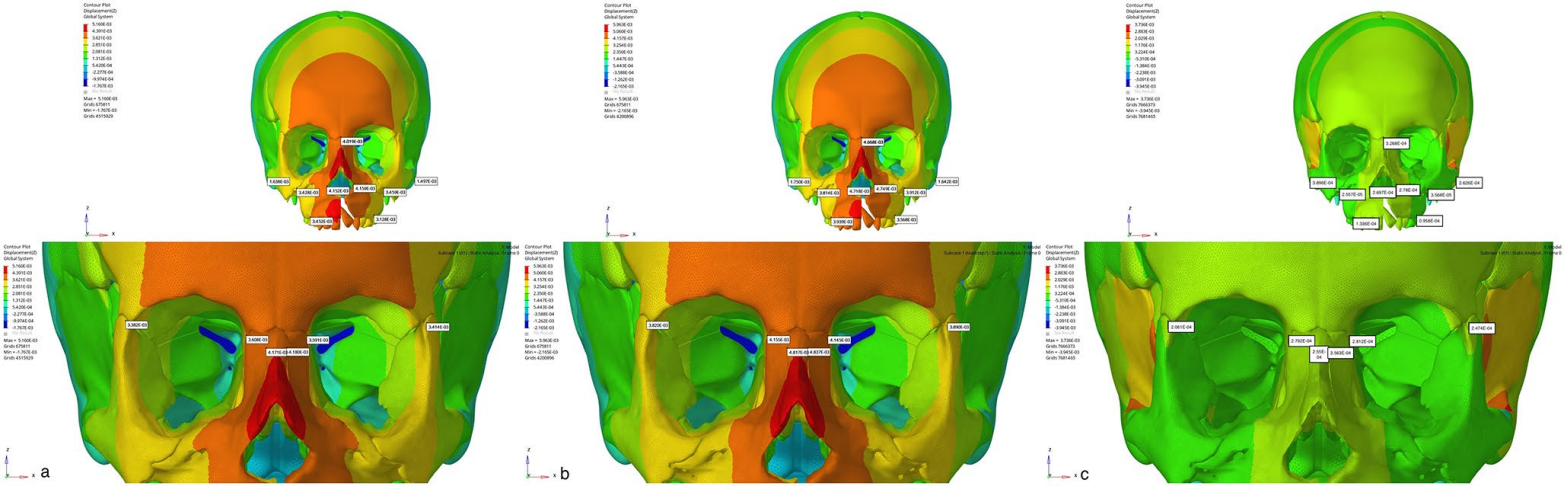
Frontal images (Z Axis) of the displacement values in the maxillary protraction models using (**a**) Face mask over acrylic plates, (**b**) face mask over infrazygomatic plates and (**c**) infrazygomatic and menton plate over elastic in the DDY model

The maximum amount of counterclockwise rotation in the maxilla as a result of protraction was observed in Group 1 and the minimum amount was observed in Group 3. As a result, a clockwise rotation of the mandible was observed in Group 1, whereas a counterclockwise rotation was observed in Group 3 (Fig.?7). The findings show that in all three models, tension forces occur in the area surrounding the maxillary posterior teeth and in the pterygomaxillary suture area, while compression forces occur in the lateral walls of the nasal region.

**Fig. 7:**
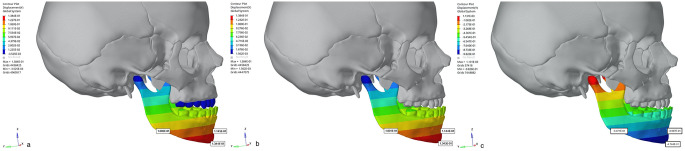
Right lateral images of the displacement values in the maxillary protraction models with the use of (**a**) Face mask over acrylic plates, (**b**) face mask over infrazygomatic plates and (**c**) elastic over infrazygomatic and menton plate in the CLP model (Y Axis)

Regarding nasomaxillary suture, Von Misses stress was observed at a minimal level from superior to inferior of the left suture in all three groups. Forces in the form of pressure on the nasomaxillary suture were more intense. When the displacement values were analyzed, a displacement to the superior direction and a compression to the posterior direction were observed in the models (Fig.?3).

Regarding frontonasal suture, frontomaxillary suture and zygomaticotemporal suture Von Misses stress showed homogeneous distribution in all three groups. When the displacement values were analyzed, displacement in the superior direction was found in the models, while slight displacement was observed posteriorly (Fig.?3). Von Misses stress in the zygomaticomaxillary suture was observed most in the inferior part of the sutural area and least in the superior area in all three models. When the displacement values were analyzed in the zygomaticomaxillary suture, an anterior movement was observed in all three groups. When Von Misses values in the zygomaticofrontal suture were analyzed, it starts intensely in the sutural area and decreases in the superior and inferior directions in all three groups. When the displacement values were analyzed, minor displacement was observed in all groups (Fig.?3).

When the maxillary dentition was taken into consideration, in Group 1, Von Misses stress was concentrated in the maxillary right first premolar and left deciduous first molar teeth, respectively. A decrease in stress was observed from the posterior region to the anterior region, and reached minimal values in the incisors. When the roots were analyzed, more stress was observed in the buccal root than in the palatal root. In the other two CLP models, since skeletal anchorage was used, the stress was homogeneously distributed with minimal values in the maxillary dentition. Looking at the displacement data in the maxillary protraction model with a face mask over a conventional acrylic plate, the crowns of the teeth in the incisor region were displaced labially, and the roots palatally when examined in the sagittal direction. In the vertical plane, there was a displacement to the superior direction in the incisors, more in the teeth adjacent to the cleft line, while it decreased towards the posterior. A similar amount of anterior displacement was observed in all teeth, and on the basis of crowns and roots, it was observed that the crowns of all teeth were displaced more anteriorly than the roots. Minor movements were observed in the skeletally supported models (Fig.?8).

**Fig. 8:**
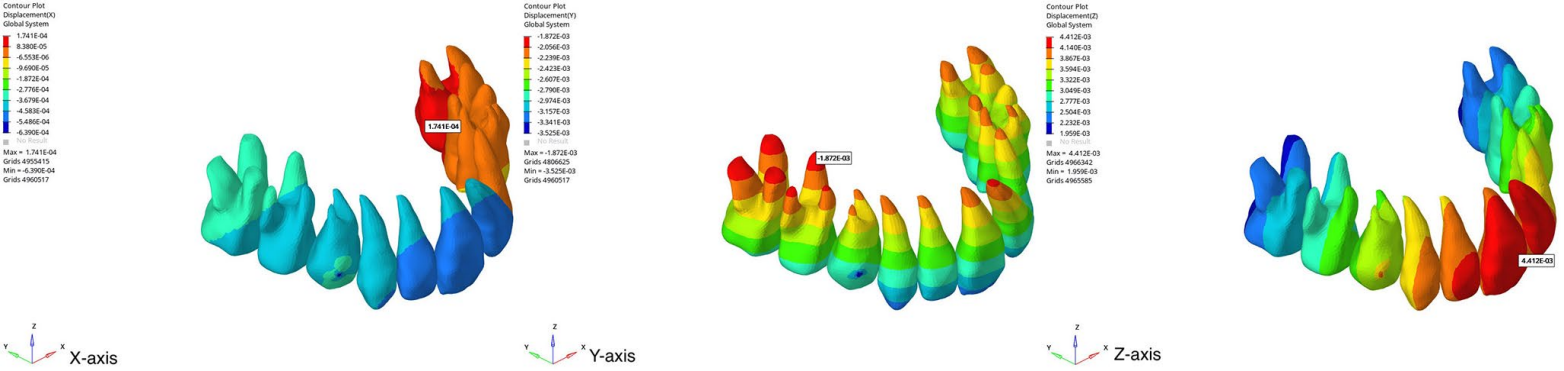
Images of the displacement values in the maxillary protraction model with a face mask over a conventional acrylic plate (x, y and z axis)

Regarding the force loading on the miniplates, the Von Misses stress continued to increase starting from the neck opening into the mouth to the level where the second miniscrew was applied. The area where the force was most concentrated was the miniplate section surrounding the inferior screw and the neck of the screw. It was observed that the miniplate and screws on the cleft side were exposed to more stress. Similarly, the Von Misses stress was concentrated mostly around the first screws of the menton plates starting from the neck opening into the mouth (Figs.?9 and 10).

**Fig. 9:**
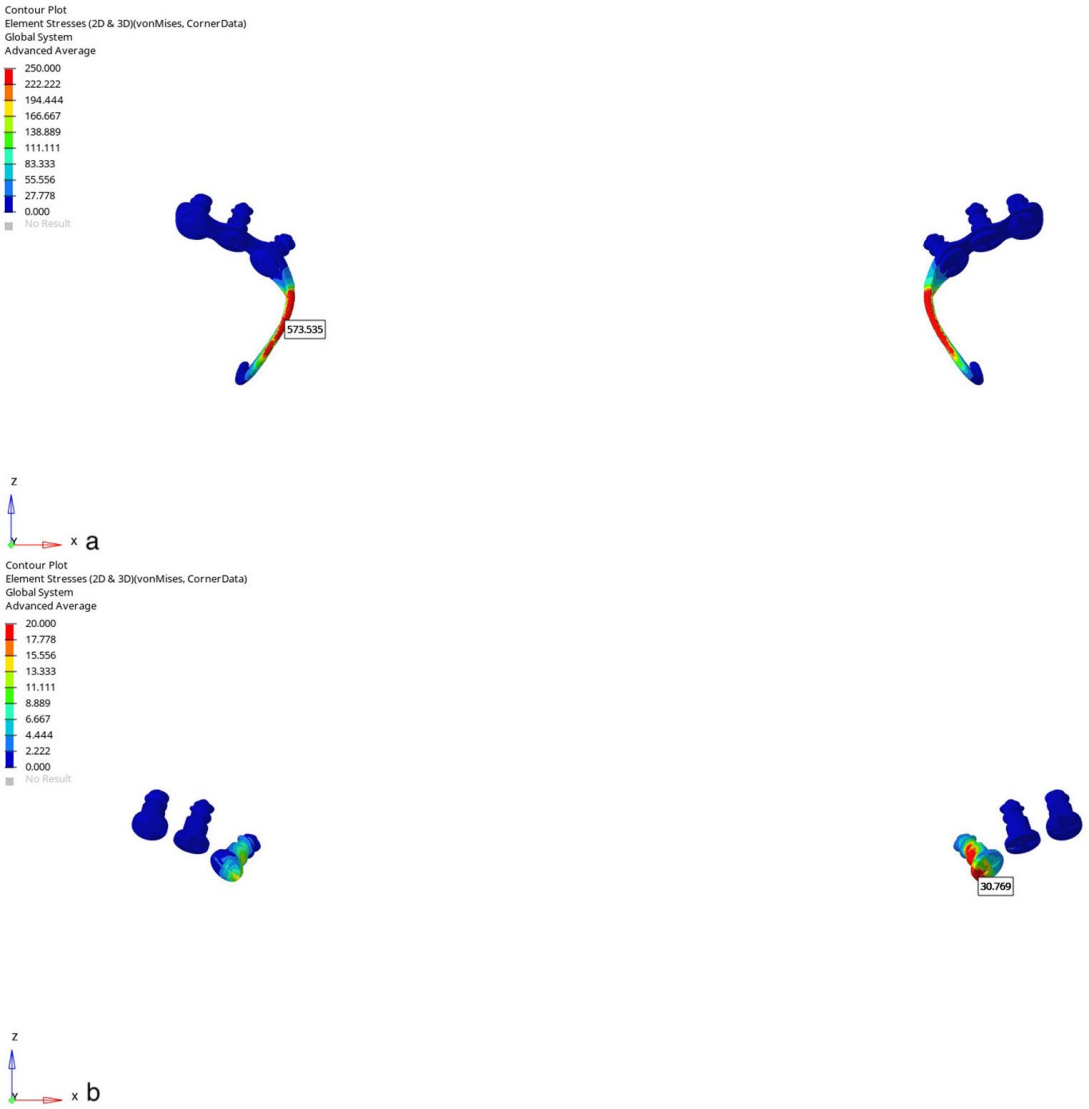
Von Misses stress images of (**a**) infrazygomatic plates and (**b**) miniscrews used in the maxillary protraction model with a face mask over infrazygomatic plates in the CLP model

**Fig. 10:**
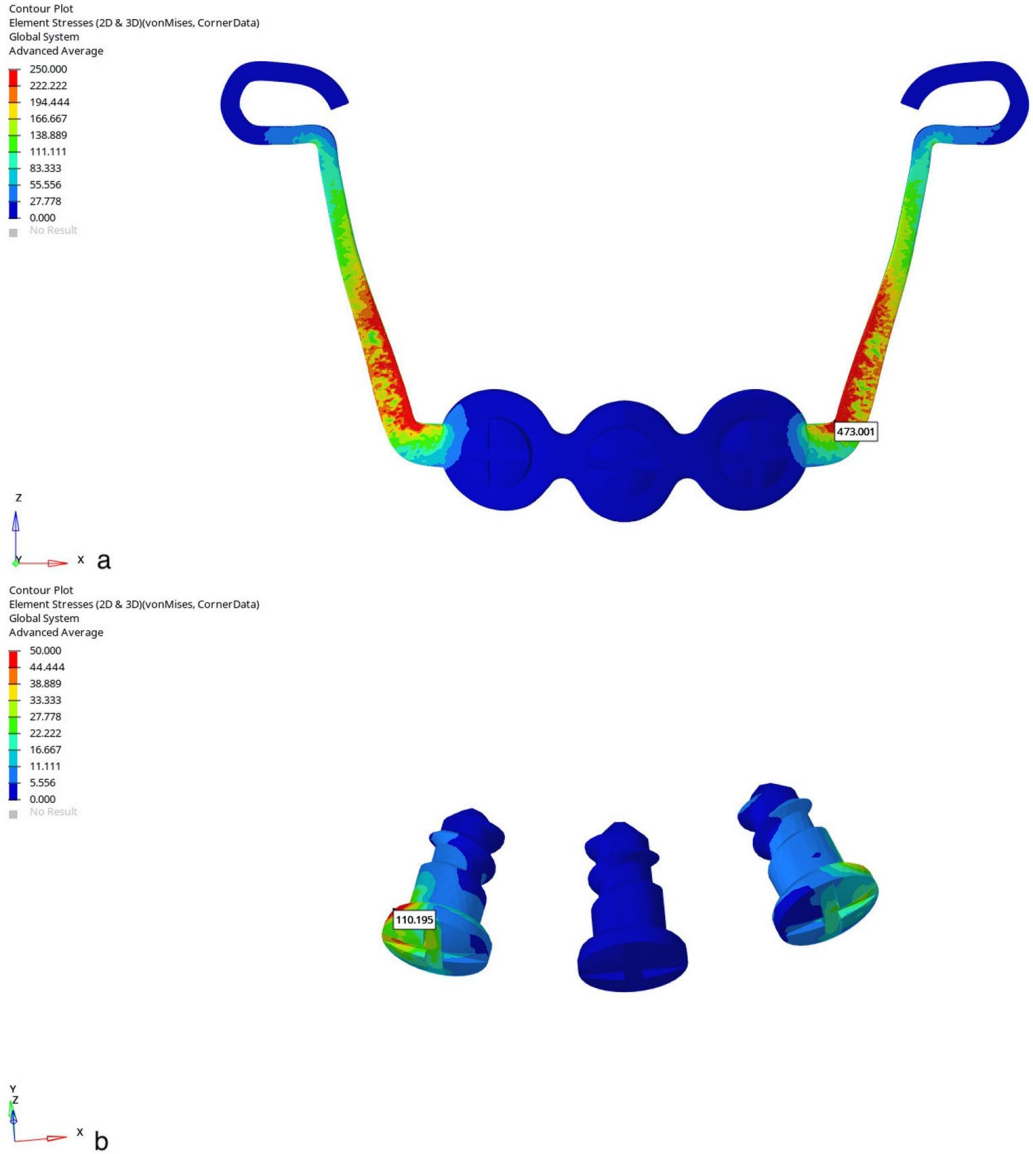
Von Misses stress images of (**a**) menton plates and (**b**) miniscrews used in the maxillary protraction model using infrazygomatic plates and elastic over menton plates in the CLP model

When the temporomandibular region is analyzed, the compression stress force is highly effective in the glenoid fossa region in Group 1 and Group 2, while it is less effective in Group 3 (Fig.?11).

**Fig. 11:**
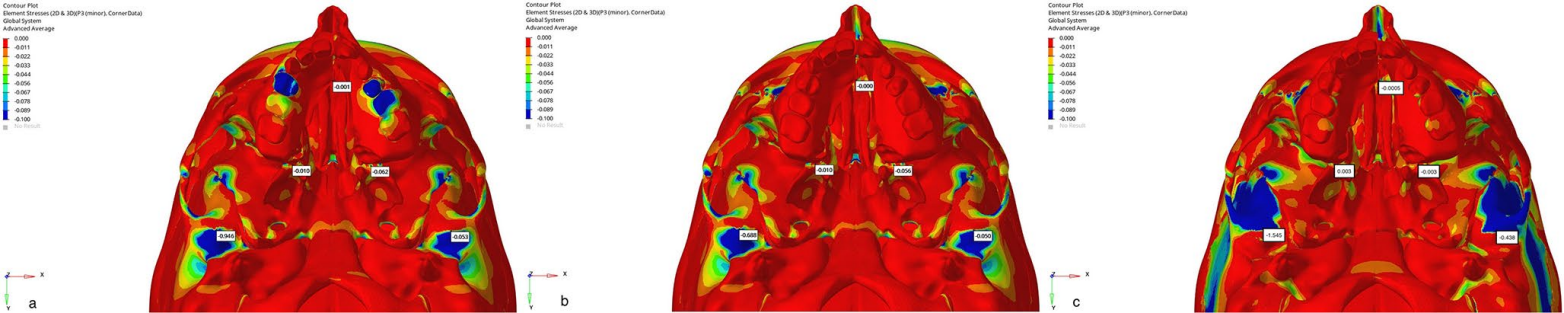
Occlusal images of minimum principal stress values in the maxillary protraction models of the CLP model using (**a**) Face mask over acrylic plates (**b**) face mask over infrazygomatic plates and (**c**) elastic over infrazygomatic and menton plate

## Discussion

Finite element analysis is a computer-aided simulation frequently used especially in engineering and health fields [17]. With these simulations, an infinite number of variables are transformed into a predictable finite number of elements. In this way, the data obtained can be converted into predictable results in terms of treatment mechanics [18]. Different methods such as modelling with medical images, anatomical modelling and segmentation have been proposed to obtain the finite element model to be analyzed [17]. In the modelling method which is frequently used in medical images, the tissues such as bone and dental structures should be well-separated and their mechanical properties should be well-defined to the program; because when the effects of the force are examined in the software, the three-dimensional image will react according to the mechanical properties and boundary values [17].

In order to create the most accurate physical features of the patient, the image used in our study was created using cone beam computed tomography data of a patient with Class III skeletal malocclusion and cleft lip and palate. The properties of structures such as enamel, dentin, cementum and periodontal ligament were manually entered to the analysis, where the reference values were applied by taking the values defined in previous studies into account [19, 20].

The acrylic plate, infrazygomatic plate, face mask, menton plate and miniscrews used in our study were modelled in ANSYS Spaceclaim software based on the dimensions in the product catalogue. The acrylic plate connected with a 0.043-inch wire passing through the palatal region was applied to open the occlusion in each model. Eman et al. [21], Yang et al. [17] and Zhang et al. [22] also used bite blocks to open the occlusion in order to correct the cross-closure in the incisor region, in their studies. On the contrary, Parveen et al. [15] did not use any appliance to open the occlusion. Routinely, acrylic plates are not used in all cases where skeletal anchorage is applied. However acrylic plates can be inserted in order to control unwanted movements of the cleft fragment, such as undesirable expansion, and to better control the movement of the alveolar bone on the cleft side.

When the literature about the forces applied for maxillary protraction in skeletal Class III maxillary retrusion patients was examined, it was seen that forces ranging from 150?g to 1200?g were applied [22?^24^]. Generally, an average force of 500?g was applied for the methods using face masks, while an average force of 150?200?g was applied for BAMP treatments. In this study, the elastic forces were applied 500?g per side for the models with face mask therapy. In the model of the elastic use over infrazygomatic and menton plates, the elastic forces were applied 250?g per side.

Not only the amount, but also the direction of the applied force is important. Parveen et al. [15] investigated the effects of face mask application with different force vectors (+?20?, 0? and ??20?) relative to the occlusal plane, on individuals with unilateral cleft lip and palate in a finite element analysis study. According to their study, it was found that more movement was obtained in all planes in patients with a face mask applied with +?20? (inferior to the occlusal plane) compared to the other groups. Although there is no general consensus in the literature regarding the angle of force application during face mask usage, it is generally preferred that the applied force should pass 20? to 30? below the occlusal plane [22, 25]. In this study, the direction of the elastic force was applied to pass 30? below the occlusal plane.

In the present study, the highest Von Misses stress in the alveolar bone adjacent to the teeth and in the lateral walls of the alveolar processes and pterygomaxillary suture as a result of the application of force was detected with a face mask over the acrylic plate appliance. This result is thought to be due to the fact that some of the force applied in the traditional model is transmitted to the dentition through the acrylic appliance on the maxillary teeth. This finding coincides with the results of previous studies that showed labial inclination of maxillary incisors following face mask treatment [26]. In the maxillary protraction model with a face mask over the infrazygomatic plates, the maximum stress occured in the lateral area of the zygomatic process of the maxillary bone, in the infrazygomatic region and in the pterygomaxillary suture. The stress, which were especially concentrated in the area where the plates were applied, gradually decreased while showing a peripheral distribution. In protraction models applied directly over the infrazygomatic crest, a slight tensile force was observed in the alveolar bone. Since the force was applied directly to the bone region in skeletally supported maxillary protraction models, it is an expected result that the stresses were concentrated in the zygomatic process region of the maxillary bone.

When the displacement values were analyzed, the highest displacement in the sagittal direction occurred in the part of the maxilla where the plates were applied and in the pterygomaxillary suture region in the skeletally supported protraction models, while in the maxillary protraction model with a face mask over acrylic plate the highest displacement was observed in the alveolar bone and surrounding structures surrounding the maxillary posterior teeth. Consistent with our findings, in an overview of systematic reviews [27], it was declared that a better anterior movement of maxilla can be expected with bone-anchored devices, in comparison with dental anchored devices during orthopaedic treatment of maxillary retrusion.

When the amount of displacement in the sagittal direction was compared, it was observed that the skeletal models showed an anterior displacement especially in the maxillary basal bone, while the maxillary protraction model with a face mask over acrylic plate showed an anterior displacement in the dentition. When the amount of displacement was evaluated, it was observed that there was more change in the maxillary protraction model with a face mask over infrazygomatic plates than in the maxillary protraction model with a face mask over an acrylic plate. The least amount of displacement was occurred in the model with menton plate compared to the other models. This is thought to be due to the fact that the angle of the application of force by the elastic is different from the other two models. Kamath et al. [24] investigated maxillary protraction by using a face mask over the infrazygomatic crest, and elastic from the infrazygomatic crest to the menton plate in their bone-assisted maxillary protraction study. They concluded that the most sagittal movement of the maxilla was found in the face mask group over the infrazygomatic crest. In this respect, our findings are in agreement with the findings of Kamath et al. [24].

In the present study, the data of a patient who had not undergone secondary alveolar bone grafting was used. In some studies [14, 22], face mask therapy performed after an alveolar bone graft produced more anterior maxillary migration and less pronounced mandibular clockwise rotation than those in the ungrafted group. It was concluded that the alveolar bone graft seemed to play a crucial role to distribute the stress more evenly between the segments. In the present study, more pronounced movement of cleft side supports the importance of secondary alveolar bone grafting on distribution of stress.

In the vertical direction (Z-axis), the maximum displacement was observed as the movement of the premaxillary region to the superior direction in the model with maxillary protraction with a face mask over infrazygomatic plates. The least movement in the vertical direction was observed in the maxillary protraction model protocol involving the use of elastic over infrazygomatic plates and menton plate. In the study conducted by Kim et al. [28], maxillary protraction model with miniplates resulted in superior movement of the maxilla, comparable with our findings. Zhang et al. [22] performed maxillary protraction with a face mask in patients with cleft lip and palate, and showed a superior movement in the anterior part of the maxilla, comparable with our study.

Although skeletally assisted maxillary protraction therapy with a face mask cannot completely prevent palatal rotation, it uses a downward and forward force. Jahanbin et al. [29] reported that a downward angle of more than 30? is needed to prevent counterclockwise rotation of the palatal plane in patients with cleft lip and palate. They stated that when intermaxillary elastics are used in the treatment of BAMP, counterclockwise rotation occurs with class III elastics. Yan et al. [30] evaluated the three-dimensional movement of the craniomaxillary complex during maxillary protraction with bone anchorage and dental anchorage by finite element analysis. In their study, the craniomaxillary complex in the dental anchorage model was displaced anteriorly with a counterclockwise rotation, and the degree of rotation gradually decreased as the angle between the force vector and the occlusal plane increased between 0? and 30?. Also, in the bone anchorage model, the craniomaxillary complex was displaced anteriorly with counterclockwise rotation and the degree of rotation gradually decreased with increasing angle from 0? to 20?. However the nasomaxillary complex rotated posteriorly with clockwise rotation when a 30? force vector was used. Similarly, in our study, there were differences in terms of elastic application between the models using face masks and the model using intermaxillary elastics. While an angle of 30 degrees was applied in the models using a face mask, an angle of 40 degrees was applied in the model where elastic was applied through miniplates. These differences affected the movements occurring in the maxilla and the degree of rotation in the maxilla. According to our findings, there was a clockwise rotation in the mandible in the models using the face mask, while there was a counterclockwise rotation in the model using elastic over miniplates, comparable with the literature [24, 29, 30]. Therefore, in cleft patients with skeletal Class III malocclusion and maxillary retrusion, lower anterior facial height can be controlled by changing the direction of the applied force, regardless of which anchorage is used.

The compressive stresses on the frontonasal, frontomaxillar and nasomaxillar sutures seem to occur as a result of the tendency for counterclockwise rotation of the nasomaxillary complex.

While the face mask over acrylic plate showed mostly dentoalveolar effect, orthopaedic effect was observed more in skeletal supported models. Especially in the zygomaticomaxillary, zygomaticotemporal and zygomaticofrontal sutures in the midface region, the stress distribution was more intense than in the tooth-supported model, and an anterior displacement was also observed. Therefore, better aesthetic results can be obtained in the mid-face region with skeletally supported models. These findings in our study are comparable with the study of Yang et al. [14].

When the displacement values in the maxillary dentition were analyzed, the findings of our study were associated with more skeletal movement in the skeletally supported maxillary advancement models and more dental movement in the acrylic plate over face mask model, comparable with the findings of Kamath et al. [24].

When the changes occurring in miniscrews with infrazygomatic plates and menton plate were analyzed, it was shown that excessive force accumulation may occur in the fixation screws used in the fixation of mini plates, especially in the screw closest to the arm in which force was applied, this may cause loss of the fixation screw. Another important consideration is the number of fixation screws used. In the study by De Clerk et al. [31], it was emphasised that the number of screws applied to the maxilla should be at least three, and three fixation screws were used in this study. If two fixation screws are used, the distribution of the force between the screws will be more critical and this will affect the effectiveness of the treatment. Additionally, it was observed that the miniplates and screws on the cleft side were subjected to more stress. This finding indicates that the risk of loss of mini screws and mini plates in that area is higher. This finding is in agreement with Jahanbin et al. [29] and Kamath et al. [24]. Kamath et al. [24] stated that the success rate could be increased with minimally invasive surgery, full compliance with postoperative instructions and good follow-up by the orthodontist.

In the light of the findings in our study, it is seen that high compression stresses occur in the glenoid fossa in the models including face mask, while less stress occurs in the maxillary protraction model including the use of elastic over infrazygomatic plates and menton plate. Stress created in the temporamandibular joint area in the early period may trigger joint disorders in the future. Mathew et al. [32] showed that the stress in the glenoid fossa decreases with the increase in the angle of the force applied for maxillary protraction with the occlusal plane in their finite element stress analysis study, which supports the findings of our study. Clinically, BAMP treatment can be preferred over face mask treatment, in patients who are prone to temporomandibular disorders.

There are some limitations of the study. Although the results obtained in this study provide information about the initial stress distribution and displacement pattern in the maxillary protraction of individuals with cleft lip and palate, the actual treatment outcome may be different because the soft tissue and post-surgical scar tissue in the lip and palate were not taken into account during modelling. Therefore, further studies are needed to investigate the effects of soft tissue and postoperative scar tissue on maxillary protraction in patients with cleft lip and palate. Furthermore, a cleft model with various cleft dimensions and maxillary complex ratio in sagittal/vertical dimensions need to be developed to generalize the results in different cleft types.

In the present study, maxillary expansion was not used in none of the maxillary advancement protocols to evaluate the effects of maxillary protraction methods, solely. However, usually maxillary expansion is the first stage before maxillary advancement protocols. In the present study, it was shown that both cleft and non-cleft sides received either expansive or contractive forces regarding the chosen maxillary advancement protocol. For example as both cleft and non-cleft sides received expansive forces during traditional face mask treatment, expanding maxilla before maxillary protraction may cause overexpansion of maxilla in patients without secondary alveolar bone graft. On the other hand, BAMP protocol resulted in narrowing of both cleft and non-cleft sides, therefore expansion can be suggested in all patients to overcome the constriction. To understand the effects of maxillary expansion further, future studies can be conducted regarding maxillary protraction methods including maxillary expansion.

## Conclusions

1. In the maxillary protraction model with a face mask over a conventional acrylic plate, dental effects were found to be greater, while skeletal effects were found to be greater in other models.
2. More movement in the sagittal plane was observed on the cleft side than the non-cleft side in all three CLP models.
3. In individuals with unilateral cleft lip and palate and Goslon score 4, the maxilla protrudes in the sagittal direction in all 3 treatment methods. The most pronounced movement is seen in face mask treatment over infrazygomatic plates.
4. Clockwise rotation of the mandible was observed in the face mask models, while counterclockwise rotation was observed in the model with infrazygomatic plates and menton plate. From a clinical point of view, BAMP protocol can be used in high-angle CLP patients.
5. It was observed that the miniplates and screws on the cleft side were exposed to more stress. This finding indicates that the risk of loss of mini screws and mini plates in that region is higher. Therefore, usage of 3 mini-screws would be more secure when stabilizing mini-plates.
6. As the angle of the force applied for maxillary protraction with the occlusal plane increases, the compressive stress in the glenoid fossa decreases.

## Abbreviations

BAMP: Bone anchored maxillary protraction
GOSLON: Great Ormond Street, London and Oslo
